# Efficacy, safety, and survival outcomes of radiotherapy combined with nimotuzumab following induction chemotherapy in locally advanced head and neck cancer

**DOI:** 10.3389/fonc.2026.1769718

**Published:** 2026-02-18

**Authors:** Yanjie Zhuang, Chen Chen, Zhenxi Xu, Kun Lin

**Affiliations:** 1 Drug Clinical Trial Facility Office, Shantou Central Hospital, Shantou, China; 2 Department of Pharmacy, Cancer Hospital of Shantou University Medical College, Shantou, China; 3 Radiotherapy Oncology Department, Shantou Central Hospital, Shantou, China; 4 Department of Public Health and Preventive Medicine, Shantou University Medical College, Shantou, China

**Keywords:** head and neck cancer, induction chemotherapy, nimotuzumab, overall survival, radiotherapy, safety, targeted therapy, treatment efficacy

## Abstract

**Objective:**

To evaluate the efficacy, safety, and impact on survival of adding nimotuzumab to radiotherapy after induction chemotherapy in patients with locally advanced head and neck cancer (HNC).

**Methods:**

This retrospective cohort study included 313 patients with locally advanced HNC treated at a tertiary hospital between 2019 and 2022. Patients were divided into an observation group (n=145), receiving radiotherapy combined with nimotuzumab after induction chemotherapy, and a control group (n=168), receiving radiotherapy alone after induction chemotherapy. Baseline characteristics, short-term efficacy (assessed using RECIST 1.1 criteria, including complete response [CR] and partial response [PR]), overall survival (OS), progression-free survival (PFS), and adverse events were compared between the two groups, and a primary analysis restricted to nasopharyngeal carcinoma (NPC) patients was performed, as NPC comprised the majority of the cohort, to more accurately assess the survival benefits of nimotuzumab in this specific subgroup. Univariate and multivariate analyses were conducted to identify prognostic factors.

**Results:**

The observation group demonstrated a significantly higher complete response rate (41.38% *vs*. 25.00%, P < 0.05) and a lower incidence of disease progression (1.38% *vs*. 5.95%, P < 0.05) compared to the control group, indicating superior short-term tumor control. In terms of long-term outcomes, the median overall survival was significantly longer in the observation group (42.3 months *vs*. 31.7 months, P = 0.008). Multivariate Cox regression analysis further confirmed nimotuzumab use as an independent protective factor for survival (HR = 0.62, 95% CI: 0.41–0.93, P = 0.021), while advanced T stage (T3–T4) and clinical stage (III–IV) were identified as independent risk factors. Notably, subgroup analysis revealed that the survival benefit of nimotuzumab was particularly pronounced in patients with nasopharyngeal carcinoma, those younger than 60 years, and those with extensive lymph node involvement (N2–N3), a finding which remained robust in a sensitivity analysis restricted to nasopharyngeal carcinoma patients. Importantly, these efficacy advantages were achieved without a significant increase in severe toxicities, as no significant differences were observed in the incidence of grade ≥3 adverse events between the two groups (P > 0.05).

**Conclusion:**

The addition of nimotuzumab to radiotherapy following induction chemotherapy significantly improves tumor response rates and long-term survival in patients with locally advanced head and neck cancer, without increasing severe treatment-related toxicity. Given that nasopharyngeal carcinoma constituted the majority of the cohort, this combination regimen represents a particularly promising therapeutic strategy for nasopharyngeal carcinoma. However, because only a small number of non-nasopharyngeal head and neck cancers were included, the observed overall survival benefit should not be generalized to non-nasopharyngeal head and neck cancers, and further prospective studies in more homogeneous populations are warranted to validate these findings.

## 1 Introduction

Head and neck cancer (HNC) is one of the most common malignancies worldwide. According to the 2018 global cancer statistics, it accounts for approximately 650,000 new cases and 330,000 deaths annually, representing a significant public health burden (1). Squamous cell carcinoma (SCC) is the predominant histological subtype. The treatment of HNC has evolved into a multidisciplinary approach involving surgery, radiotherapy (RT), and systemic therapy (2). For patients with locally advanced disease, therapeutic objectives extend beyond local control to improving long-term survival outcomes.

Induction chemotherapy (IC) plays a critical role in the management of locally advanced HNC. Numerous studies have demonstrated that regimens such as docetaxel, cisplatin, and fluorouracil (TPF) can significantly enhance tumor response rates, thereby facilitating subsequent definitive radiotherapy (3, 4). However, a phase III trial by Cohen et al. revealed that a considerable proportion of patients still experienced disease progression after IC followed by radiotherapy alone, highlighting the limitations of this sequential approach (5). This clinical challenge has spurred interest in developing combination strategies to enhance radiosensitivity and improve local control.

The advent of molecularly targeted therapies has fundamentally reshaped the treatment landscape for head and neck carcinoma. A key molecular feature is the frequent overexpression of the epidermal growth factor receptor (EGFR), observed in over 90% of cases. The constitutive activation of EGFR-mediated signaling pathways plays a central role in tumorigenesis, progression, invasion, metastasis, and resistance to conventional therapies (6). Consequently, anti-EGFR monoclonal antibodies have emerged as an important direction in targeted therapy for HNC. Nimotuzumab is a humanized IgG1 monoclonal antibody specifically designed to target EGFR. It binds selectively to the extracellular domain of EGFR, blocking ligand-induced activation and thereby inhibiting downstream signaling (7).

Preclinical and clinical studies strongly support the use of nimotuzumab. Through structural biology research, Talavera et al. elucidated its mechanism of action, demonstrating that it inhibits ligand binding while preserving the active conformation of the receptor (7). Crombet et al. provided the foundational clinical evidence by reporting the efficacy and safety of combining nimotuzumab with radiotherapy in locally advanced HNC (8). Subsequent phase I trials further confirmed the feasibility of this combination (9). Importantly, compared with cetuximab, nimotuzumab exhibits a more favorable safety profile, particularly with regard to a significantly lower incidence of severe skin toxicity (10)—an advantageous feature for patients requiring prolonged treatment.

Current research on nimotuzumab in HNC primarily focuses on its combination with concurrent chemoradiotherapy (11, 12). However, evidence supporting its integration into the sequential model of “induction chemotherapy followed by radiotherapy” remains limited. Theoretically, while IC reduces tumor burden, it may select for more aggressive tumor subclones. The addition of an EGFR inhibitor with radio sensitizing properties at this stage could more effectively eradicate residual tumor cells and overcome therapeutic resistance (13). This biologically rational sequential strategy warrants further validation in clinical practice.

Against this background, this retrospective cohort study aims to comprehensively evaluate the short-term tumor response, long-term survival, and safety profile of a treatment regimen combining nimotuzumab with radiotherapy after induction chemotherapy in patients with locally advanced head and neck cancer. The findings are intended to provide robust clinical evidence supporting the use of this integrated therapeutic approach.

## 2 Materials and methods

### 2.1 Inclusion and exclusion criteria

Inclusion Criteria: (1) Patients with pathologically confirmed HNC via biopsy, newly diagnosed; (2) Patients meeting the indications for radiotherapy; (3) Age ≥16 years; (4) Absence of acute infection or severe systemic comorbidities.

Exclusion Criteria: (1) Prior use of EGFR inhibitor or similar drugs before planned concurrent chemoradiotherapy; (2) Prior surgical resection of the primary tumor or involved lymph nodes before radiotherapy or induction chemotherapy; (3) Radiotherapy interruption due to any reason; (4) History of other malignancies or diagnosis of double primary tumors; (5) Patients with non-evaluable efficacy. This study was approved by the hospital’s Ethics Committee (Approval No.: 2022-Science-091).

### 2.2 Baseline data collection

This study collected clinical data from 313 HNC patients diagnosed and treated at a tertiary hospital between 2019 and 2022. Follow-up ended at patient death or February 2024. Collected data included gender, age, pathological subtype, HNC type, T stage, N stage, M stage, and clinical stage, and the cohort comprised both nasopharyngeal carcinoma and non-nasopharyngeal head and neck cancer, with nasopharyngeal carcinoma constituting the majority of cases.

### 2.3 Treatment methods

Induction Chemotherapy (IC): The interval between IC and radiotherapy (RT), treatment delays, and patient compliance were carefully monitored and recorded to assess their impact on treatment outcomes. The time gap between IC and RT was kept within standard guidelines, and any deviations from the protocol, including delays, were documented. Patients received one of three induction chemotherapy regimens: 1) The TP regimen, consisting of paclitaxel liposome (135–175 mg/m^2^) administered on day 1, combined with either cisplatin (30 mg/m^2^ on days 1–3) or nedaplatin (80 mg/m^2^ on day 1); 2) The DP regimen, which included docetaxel (75 mg/m^2^ on day 1) along with the same cisplatin or nedaplatin options; 3) The TPF regimen, comprising docetaxel (60 mg/m^2^ on day 1), fluorouracil (600 mg/m^2^ on days 1–5), and the aforementioned cisplatin or nedaplatin schedule.

Radiotherapy: All patients received standard intensity-modulated radiotherapy (IMRT). The specific procedure was: (1) Patient positioning in the supine position with a mouthpiece, head resting on a customized foam pillow, and immobilized using a thermoplastic mask covering the head, neck, and shoulders. (2) CT simulation scan in the supine position, scanning from the vertex to 2 cm below the sternal notch via spiral CT (plain + contrast-enhanced), with a slice thickness of 3 mm. (3) Contouring of target volumes and organs at risk (OARs). MRI or CT was performed after induction chemotherapy and before starting radiotherapy to assess the tumor response and define the gross tumor volume (GTV). This allowed for precise planning of subsequent radiotherapy. Additionally, target volumes, including GTV and clinical target volumes (CTV), were delineated using pre-induction chemotherapy MRI with both non-contrast and contrast-enhanced sequences. The GTV was defined for the primary tumor and involved lymph nodes, while the CTV included areas at high risk for subclinical disease spread. OARs, such as the optic nerves, optic chiasm, lenses, temporal lobes, brainstem, spinal cord, inner ears, parotid glands, and temporomandibular joints, were delineated on the simulation computed tomography (CT) images. GTV-nx: Primary tumor region, including involved retropharyngeal lymph nodes. GTV-nd: Cervical metastatic lymph nodes. CTV-60 (High-risk clinical target volume): Area with high probability of subclinical spread around the primary site, defined as GTV-nx with a 5–10 mm margin, plus the entire nasopharyngeal mucosa and a 5 mm superior-inferior margin. CTV-54 (Low-risk clinical target volume): Area at potential risk of involvement and prophylactic neck nodal regions, including the clivus, parapharyngeal space, posterior nasopharynx, pterygopalatine fossa, etc., and a 5–10 mm expansion from CTV-60 in other directions. PTV: Planning target volumes created by expanding CTV-60 and CTV-54 by 3–5 mm in anterior, superior, inferior, left, and right directions, and 2–3 mm posteriorly, forming PTV-60 and PTV-54, respectively. The prescribed doses were as follows: PGTV-nx received 68–70 Gy, PGTV-nd received 60–70 Gy, PTV-60 received 60 Gy, and PTV-54 received 54 Gy, all delivered in 30 fractions. Treatment interruptions were recorded, and any delays in radiotherapy were documented and managed to minimize their impact on treatment efficacy. Additionally, adaptive radiotherapy replanning was performed in cases of significant tumor shrinkage during treatment to ensure optimal target volume coverage and minimize exposure to surrounding normal tissues. Dose-volume constraints for organs at risk adhered to the Radiation Therapy Oncology Group (RTOG) 0615 and 0225 guidelines.

Targeted Therapy: Patients in the observation group received concurrent nimotuzumab with radiotherapy. Nimotuzumab was administered at 200 mg per dose, diluted in 250 ml of 0.9% sodium chloride solution, and delivered via intravenous infusion. Targeted therapy was given once weekly for a total of 6 doses. Patients with distant metastases received systemic therapies, such as chemotherapy or targeted therapy. Local therapies, including radiotherapy, were considered for oligometastatic disease. Adaptive radiotherapy replanning was performed in cases of significant tumor shrinkage during treatment, ensuring optimal coverage of target volumes while minimizing exposure to surrounding normal tissues.

### 2.4 Evaluation criteria

Imaging results within one month before IC served as the baseline for efficacy evaluation. At 12 weeks after completion of radiotherapy, follow-up imaging was performed. Efficacy was assessed independently by two radiologists blinded to treatment allocation. In cases of discrepancy, a consensus was reached after joint discussion. Response was evaluated using Response Evaluation Criteria in Solid Tumors version 1.1 (RECIST 1.1) (8).

The therapeutic response was evaluated according to the following criteria:

1. Complete Response (CR): Disappearance of all measurable target lesions, with any pathological lymph nodes reduced to a short-axis diameter of less than 10 mm, maintained for at least four weeks.
2. Partial Response (PR): A reduction of at least 30% in the sum of diameters of target lesions, maintained for at least four weeks.
3. Stable Disease (SD): Changes in tumor measurements not meeting the criteria for PR or PD.
4. Progressive Disease (PD): An increase of at least 20% in the sum of diameters of target lesions, or the appearance of new lesions.

To further assess the association between tumor shrinkage and prognosis, Depth of Response (DpR) was evaluated based on the maximum percentage reduction in the sum of target lesion diameters from baseline to the end of radiotherapy. Patients were classified into three groups:

- Deep Response: CR or ≥60% reduction in target lesion diameter, with pathological lymph nodes <10 mm, sustained for ≥4 weeks.
- Moderate Response: Reduction of 30–59% in target lesion diameter, sustained for ≥4 weeks.
- Stable Disease/Progressive Disease (SD/PD): Reduction of <30%, any increase, or appearance of new lesions.

The primary endpoint of the study was Overall Survival (OS), defined as the time from treatment initiation to death from any cause. Secondary endpoints included Progression-Free Survival (PFS), Objective Response Rate (ORR), and the safety profile of the treatment regimen.

Safety Assessment: All treatment-emergent adverse events (TEAEs) of grade 3 or above, deemed attributable to the study interventions and occurring either during the active treatment phase or within the 90-day post-treatment follow-up window, were systematically recorded and graded in accordance with the National Cancer Institute Common Terminology Criteria for Adverse Events (NCI CTCAE), version 5.0. In addition, late treatment-related toxicities (assessed ≥3 months post-radiotherapy) were also collected and graded using the same CTCAE criteria. Any-grade toxicities, including nimotuzumab-specific events such as skin rash, infusion-related reactions, and constitutional symptoms, were also documented and descriptively summarized. Quality-of-life outcomes were evaluated based on patient-reported data obtained from the EORTC QLQ-C30 and QLQ-H&N35 questionnaires administered at 6 and 12 months after treatment.

Response assessment after radiotherapy was performed using standard imaging techniques. For residual lesions, metabolic imaging (such as PET-CT) was not routinely performed. However, clinical evaluation and follow-up imaging, including MRI or CT, were used to monitor for progression or resolution. Locoregional control was assessed by monitoring the absence of progression in the primary tumor and regional lymph nodes. Follow-up imaging, including MRI and CT scans, was conducted at regular intervals to detect locoregional recurrence and assess the effectiveness of radiotherapy.

### 2.5 Statistical analysis

Data were entered into Microsoft Excel 2010 and analyzed using SPSS version 21.0. Continuous variables are presented as mean ± standard deviation (x̄ ± s) and compared using the Student’s t-test. Categorical variables are presented as frequencies (percentages) and compared using the Chi-square (χ^2^) test. For ordinal data, the non-parametric Mann-Whitney U test was used. Binary logistic regression analysis was employed to identify factors associated with survival prognosis, with T stage, N stage, clinical stage, and treatment modality as independent variables (Xi) and survival status as the dependent variable (Y). A two-sided P-value ≤ 0.05 was considered statistically significant. Given the limited number of death events relative to the number of covariates, the logistic regression model may be prone to overfitting, and its results should therefore be interpreted with caution as exploratory.

Survival Statistical Analysis: Kaplan-Meier survival curves were generated to estimate survival probabilities in the overall cohort and in the nasopharyngeal carcinoma subgroup, and intergroup comparisons were performed using the log-rank test. To determine independent factors influencing survival, both univariate and multivariate analyses were conducted via Cox proportional hazards regression models, which provided hazard ratios (HR) with 95% confidence intervals (CI). The multivariate model was adjusted for age, gender, T-stage, N-stage, clinical stage, histological type of HNC, treatment regimen, and induction chemotherapy regimen.

To explore the consistency of nimotuzumab efficacy across different patient populations, pre-specified subgroup analyses were conducted based on baseline characteristics (age, HNC type, T stage, N stage, clinical stage). Interaction tests were performed to assess the heterogeneity of treatment effects across subgroups.

Differences in overall survival OS across DpR subgroups were assessed using Kaplan-Meier analysis and the log-rank test. The independent prognostic significance of DpR for OS was examined through a multivariate Cox regression model that accounted for potential confounding variables, including age, gender, stage, and treatment modality.

## 3 Results

### 3.1 Baseline characteristics of the observation and control groups

**Figure 1:**
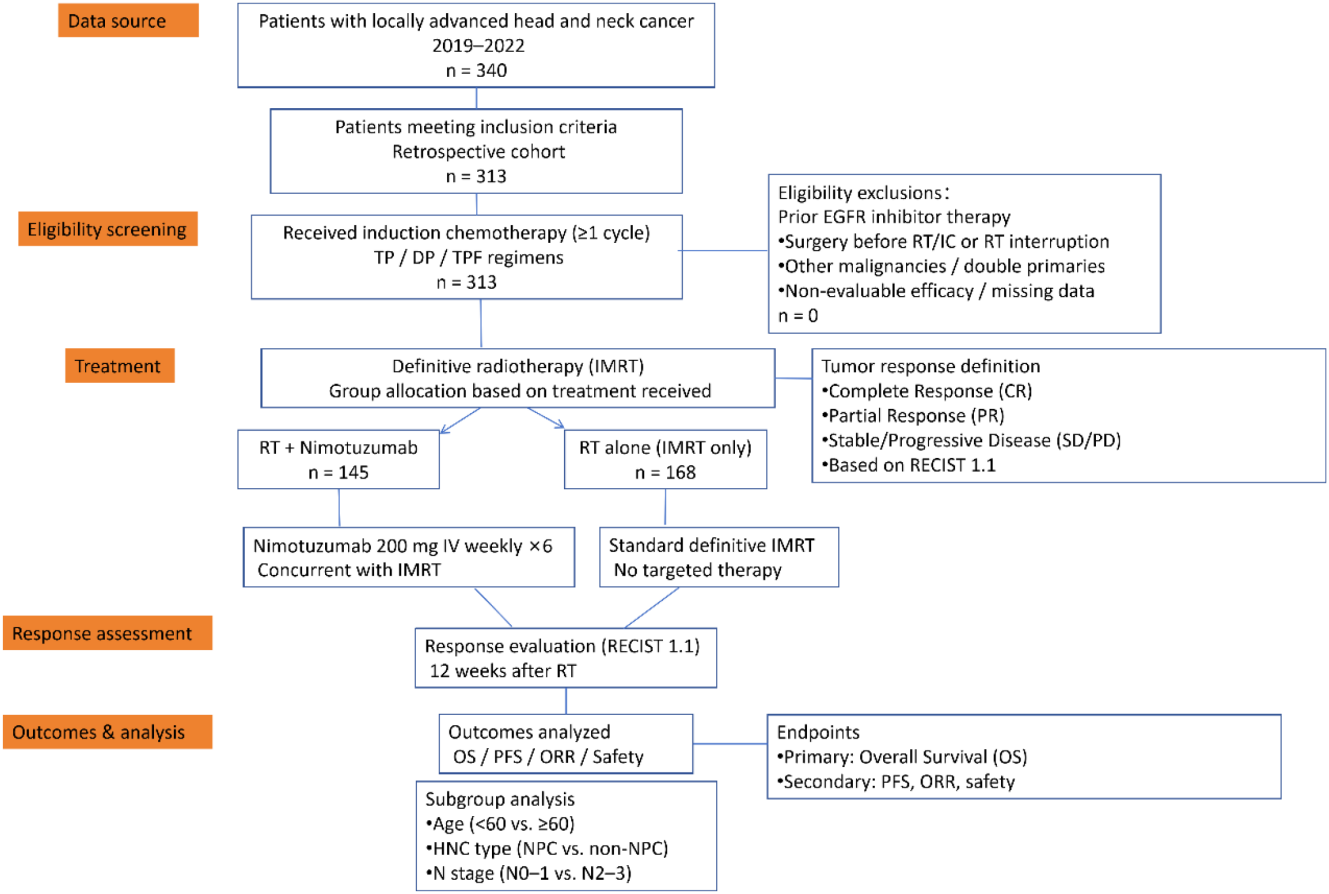
Study flowchart.

A comparative analysis of patient characteristics demonstrated balanced distribution between groups for sex, T stage, M stage, and clinical stage (all p > 0.05). Although statistical analysis showed imbalances in age, pathological classification, tumor subtype, and N stage between the two groups, these factors, particularly in NPC patients, may heavily influence the observed survival benefit. A more detailed stratification of NPC patients, considering these confounding factors, was performed to minimize the impact of these baseline differences (**Table 1**). The distribution of induction chemotherapy regimens (TP, DP, and TPF) was comparable between the two groups. In the observation group, 60 patients (41.4%) received the TP regimen, 42 (29.0%) received DP, and 43 (29.7%) received TPF. In the control group, 67 patients (39.9%) received TP, 52 (31.0%) received DP, and 49 (29.2%) received TPF. No significant differences were observed in regimen distribution between the two groups (P = 0.96). The study flowchart is shown in **Figure 1**.

**Table 1:** Comparison of baseline characteristics between the observation group and control group (n, %).

Characteristic	Category	Observation Group (n=145)	Control Group (n=168)	t/χ^2^ Value	P value
Gender, n (%)	Male	107 (73.79)	135 (80.36)	1.912	0.167
	Female	38 (26.21)	33 (19.64)		
Age, years		51.43 ± 13.11	56.48 ± 11.10	-3.689	**0**
Pathological Subtype, n (%)	Undifferentiated non-keratinizing carcinoma	121 (83.45)	111 (66.07)	22.325	**0**
	Differentiated non-keratinizing carcinoma	12 (8.28)	14 (8.33)		
	Keratinizing carcinoma	4 (2.76)	3 (1.79)		
	Squamous cell carcinoma	8 (5.51)	40 (23.81)		
HNC Type, n (%)	Nasopharyngeal carcinoma	138 (95.17)	130 (77.38)	20.012	**0**
	Non-nasopharyngeal carcinoma	7 (4.83)	38 (22.62)		
T Stage, n (%)	T0	0 (0.00)	5 (2.98)	8.574	0.073
	T1	16 (11.03)	22 (13.10)		
	T2	35 (24.14)	38 (22.62)		
	T3	52 (35.86)	47 (27.98)		
	T4	42 (28.97)	56 (33.32)		
N Stage, n (%)	N0	10 (6.90)	33 (19.64)	15.259	**0.0016**
	N1	32 (22.07)	43 (25.60)		
	N2	77 (53.10)	77 (45.83)		
	N3	26 (17.93)	15 (8.92)		
M Stage, n (%)	M0	127 (87.59)	147 (87.50)	0.001	0.982
	M1	18 (12.41)	21 (12.50)		
Clinical Stage, n (%)	I	4 (2.76)	5 (2.98)	5.968	0.113
	II	8 (5.52)	22 (13.10)		
	III	69 (47.59)	67 (39.88)		
	IV	64 (44.13)	74 (44.04)		

Bold values indicate statistical significance (P < 0.05).

Statistically significant differences between groups in age, pathological classification, tumor subtype, and N stage (p < 0.05) may introduce potential confounders, particularly in nasopharyngeal carcinoma patients.

### 3.2 Comparison of overall efficacy

In the observation group, CR was achieved in 41.38% of patients, PR in 53.79%, SD in 3.45%, and PD in 1.38%. At the final follow-up, 86.90% of patients were alive and 13.10% had died from any cause. In the control group, the rates were as follows: CR 25.00%, PR 58.33%, SD 10.71%, and PD 5.95%, with 77.98% alive and 22.02% deceased. These overall survival and mortality rates reflect all-cause outcomes over the entire follow-up period and should not be interpreted as representing acute treatment-related mortality. The absolute between-group differences were: CR + 16.38%, PR –4.54%, SD –7.26%, PD –4.57%, overall survival +8.92%, and mortality –8.92%.

Comparative analysis of treatment efficacy demonstrated a significantly higher CR rate in the observation group compared to the control group (41.38% *vs*. 25.00%, P = 0.001), along with notably lower rates of SD and PD (**Table 2**). Although no significant difference was observed in PR between the two groups (P > 0.05), the shift from non-response (SD/PD) to complete response suggests improved tumor eradication with the addition of nimotuzumab.

**Table 2:** Comparison of overall efficacy between the two groups (n, %).

Group	n	Complete response	Partial response	Stable disease	Progressive disease	Survival rate	Mortality rate
Observation Group	145	60 (41.38)	78 (53.79)	5 (3.45)	2 (1.38)	126 (86.90)	19 (13.10)
Control Group	168	42 (25.00)	98 (58.33)	18 (10.71)	10 (5.95)	131 (77.98)	37 (22.02)
Z/χ^2^ Value		-3.248				4.216	
p Value		**0.001**				**0.04**	

Bold values indicate statistical significance (P < 0.05).

Survival rate and mortality rate refer to overall all-cause survival status at last follow-up, and do not represent acute treatment-related mortality.

Furthermore, the objective response rate (ORR, defined as CR + PR) was significantly higher in the observation group than in the control group (95.17% [138/145] *vs*. 83.33% [140/168], P ≈ 0.001), indicating an overall enhancement of antitumor activity with the combined regimen.

It should be noted that the absolute CR rates observed in both groups were lower than those typically reported for homogeneous NPC cohorts treated with IMRT, where CR rates often range between 80% and 95%. This discrepancy may be attributed to the inclusion of non-NPC head and neck cancers in our study cohort, the relatively early timing of response assessment at 12 weeks after radiotherapy completion, and the application of stringent RECIST 1.1 criteria, which may underestimate late radiographic resolution of residual lesions in locally advanced disease.

### 3.3 Comparison of baseline characteristics between the survival and death groups

Based on their final survival status, patients were stratified into two distinct cohorts: a Survival Group and a Death Group. Among the 313 patients, 56 (17.89%) had died. There were no significant differences between the two groups in terms of gender, age group, pathological subtype, HNC type, or T stage (P > 0.05). Significant differences were observed in N stage, clinical stage, and treatment modality (P < 0.05) (**Supplementary Table S1**).

### 3.4 Logistic regression analysis for survival prognosis

Using survival status as the dependent variable and T stage, N stage, clinical stage, and treatment modality as independent variables (**Supplementary Table S2**), the logistic regression analysis revealed that treatment with radiotherapy combined with nimotuzumab following induction chemotherapy was a significant protective factor for survival prognosis in HNC patients (P < 0.05). In contrast, T2 stage, T3 stage, and clinical stage II were identified as significant risk factors (P < 0.05) (**Supplementary Table S3**).

### 3.5 Survival analysis and prognostic model evaluation

#### 3.5.1 Kaplan-Meier survival curve analysis

**Figure 2:**
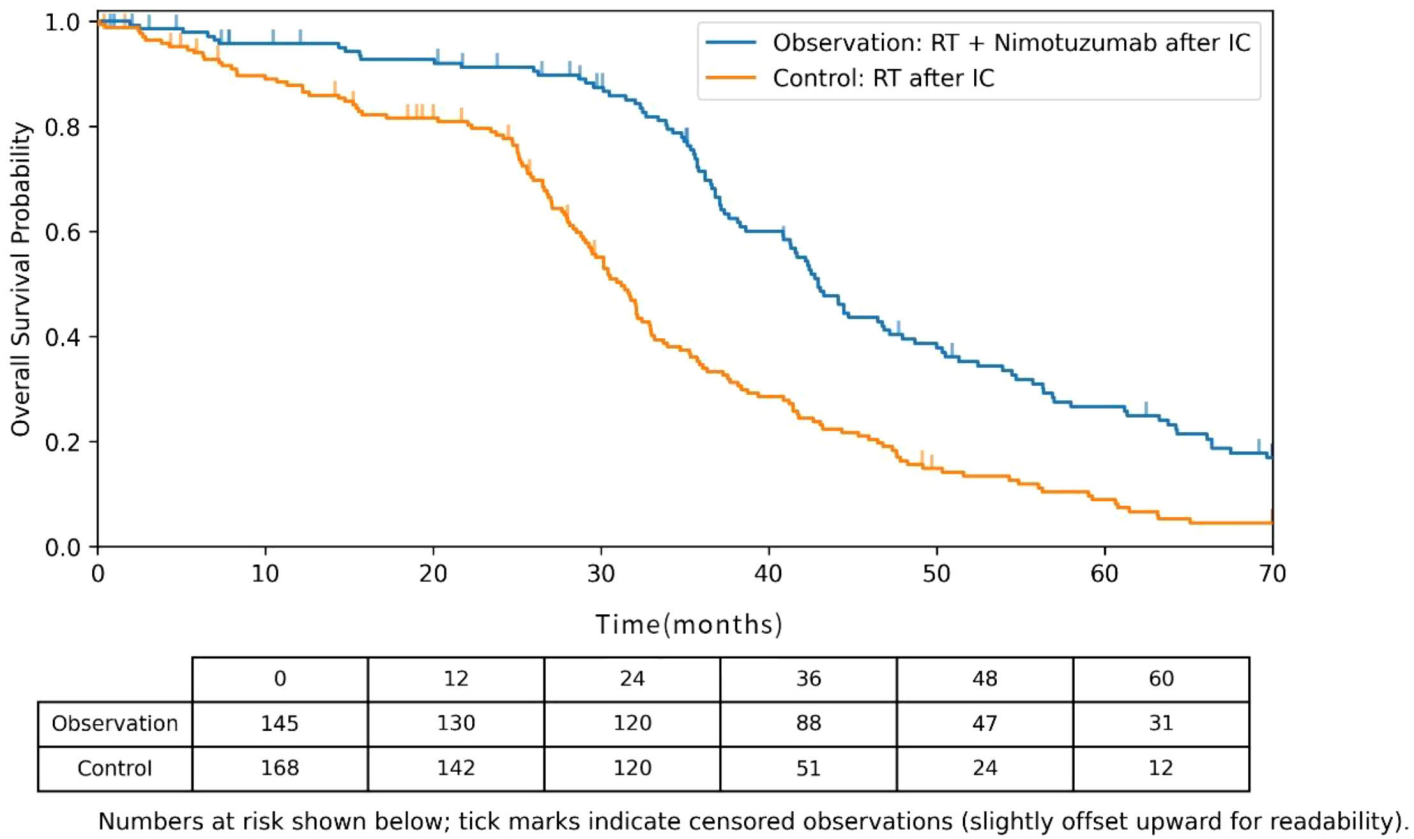
Kaplan-Meier overall survival (OS) curves for the observation group (radiotherapy + nimotuzumab after induction chemotherapy) and the control group (radiotherapy after induction chemotherapy).

To further evaluate the impact of treatment modality on long-term survival, Kaplan-Meier OS curves were generated for the overall cohort and for the NPC subgroup (**Figure 2**). The mOS was significantly longer in the observation group (radiotherapy plus nimotuzumab after induction chemotherapy) than in the control group (radiotherapy alone after induction chemotherapy): 42.3 months (95% CI: 38.5–46.1) versus 31.7 months (95% CI: 28.4–35.0), Log-rank P = 0.008. The 2-year OS rate was also significantly higher in the observation group compared with the control group (89.7% *vs*. 78.6%, P = 0.012). These findings are consistent with evidence from prior multicenter randomized controlled trials in locoregionally advanced nasopharyngeal carcinoma, in which the addition of induction chemotherapy to chemoradiotherapy improved failure-free survival (3-year FFS 80% *vs*. 72%, HR = 0.68). Together, these results support the conclusion that an intensified comprehensive treatment approach enhances long-term outcomes in this population.

#### 3.5.2 Multivariate Cox regression analysis

**Figure 3:**
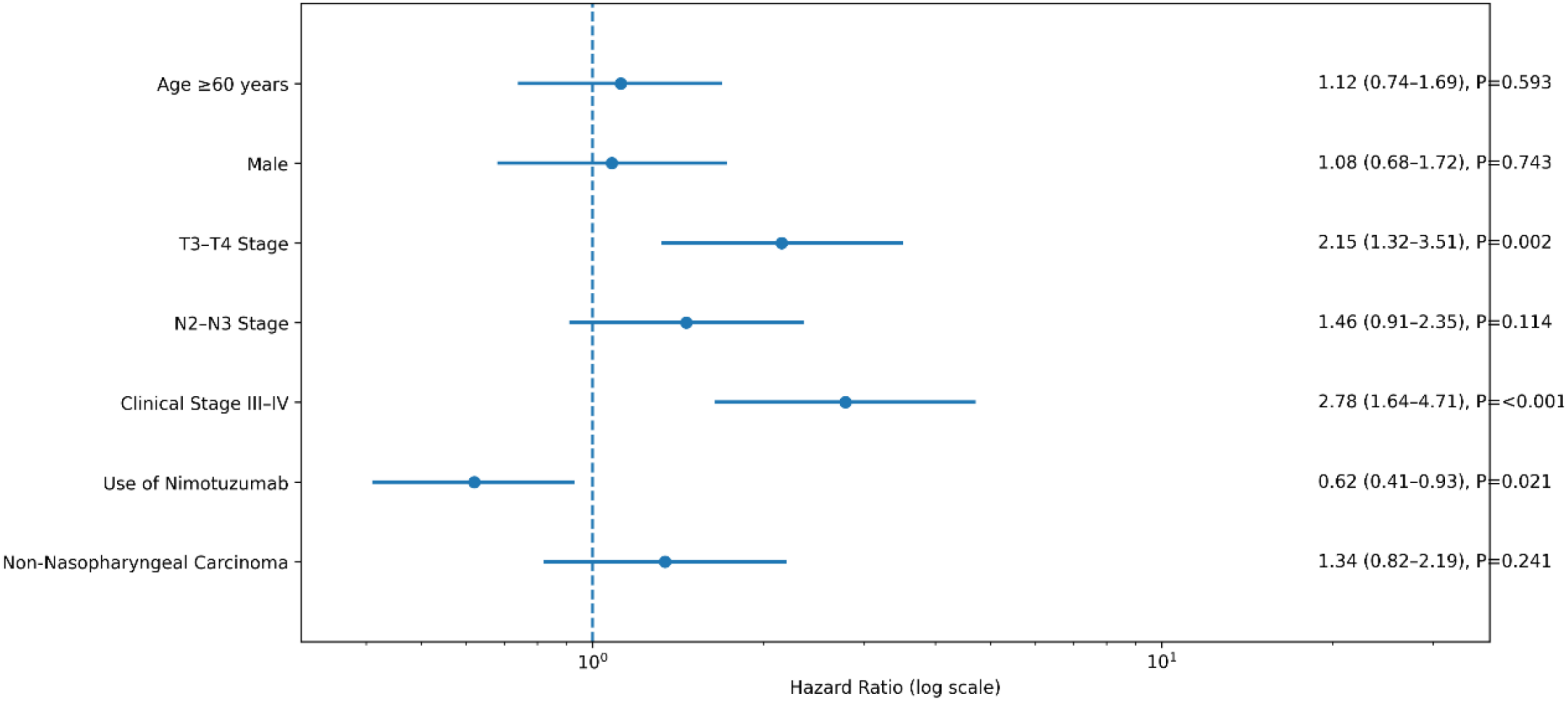
Forest plot of the multivariate Cox regression analysis for overall survival in head and neck cancer patients.

A multivariate Cox proportional hazards regression analysis was conducted to control for potential confounding variables, with adjustments made for patient age, sex, TNM stage, histopathological subtype, and treatment strategy (**Table 3**). The analysis revealed that the use of nimotuzumab served as an independent protective factor associated with improved survival. Conversely, advanced local tumor stage (T3–T4) and late overall clinical stage (III–IV) were both identified as significant independent risk factors for mortality. The summarized results of this multivariate analysis are presented in a forest plot (**Figure 3**).

**Table 3:** Multivariate Cox regression analysis of factors affecting overall survival in head and neck cancer patients.

Variable	HR	95% CI	P value
Age ≥60 years	1.12	0.74 – 1.69	0.593
Male	1.08	0.68 – 1.72	0.743
T3–T4 Stage	2.15	1.32 – 3.51	0.002
N2–N3 Stage	1.41	0.91 – 2.35	0.165
Clinical Stage III–IV	2.78	1.64 – 4.71	<0.001
Use of Nimotuzumab	0.62	0.41 – 0.93	0.021
Non-Nasopharyngeal Carcinoma	1.34	0.82 – 2.19	0.241

#### 3.5.3 Adverse reactions and safety analysis

**Table 4** details the frequency of treatment-related adverse events graded 3 or higher. Statistical analysis revealed no significant differences between the observation and control groups for grade ≥3 oral mucositis, radiation dermatitis, leukopenia, thrombocytopenia, or liver function abnormalities (all P > 0.05). This suggests that the addition of nimotuzumab to induction chemotherapy followed by radiotherapy did not significantly increase the risk of severe acute adverse events.

**Table 4:** Comparison of grade ≥3 treatment-related adverse events between the two groups (n, %).

Adverse event	Observation group (n=145)	Control group (n=168)	P value
Oral Mucositis	28 (19.3%)	33 (19.6%)	0.943
Radiation Dermatitis	19 (13.1%)	21 (12.5%)	0.873
Leukopenia	36 (24.8%)	40 (23.8%)	0.834
Thrombocytopenia	14 (9.7%)	17 (10.1%)	0.896
Liver Dysfunction	8 (5.5%)	10 (6.0%)	0.857

Beyond grade ≥3 events, the overall pattern of any-grade toxicities was also comparable between groups (data not shown), with the majority of events being grade 1–2 mucositis, dermatitis, hematologic abnormalities, and transient liver dysfunction. Nimotuzumab-specific adverse events, such as acneiform rash and infusion-related reactions, were predominantly grade 1–2, did not lead to permanent treatment discontinuation, and no anaphylactic reactions were observed. Importantly, no deaths were adjudicated as directly attributable to acute toxicity from radiotherapy or nimotuzumab; most deaths during follow-up were related to disease progression, as reflected in the overall survival analysis.

#### 3.5.4 Long-term late toxicity, quality of life, and emotional distress in the IMRT era for NPC

Late treatment-related toxicity data were available for 178 patients (92 in the observation group and 86 in the control group) who completed at least 12 months of follow-up, representing a subset of the overall cohort. The most commonly observed late toxicities included xerostomia, dysphagia, and subcutaneous fibrosis. There were no statistically significant differences in the incidence of grade ≥2 late toxicities between the two groups: grade ≥2 xerostomia occurred in 23.9% of patients in the observation group versus 25.6% in the control group (P = 0.78); grade ≥2 dysphagia in 14.1% *vs*. 15.1% (P = 0.85); and subcutaneous fibrosis in 9.8% *vs*. 11.6% (P = 0.69), respectively.

In terms of QoL outcomes, structured QoL assessments using the EORTC QLQ-C30 and QLQ-H&N35 questionnaires were retrospectively available for 102 patients. Global health status scores and key functional domains (swallowing, speech, and social eating) showed no statistically significant difference between groups at 6 and 12 months post-treatment. Within the constraints of this incomplete and relatively short-term follow-up, we did not observe a clear signal of increased late toxicity or deterioration in patient-reported quality of life associated with the addition of nimotuzumab; however, these exploratory observations are based on a subset of patients and should not be interpreted as definitive evidence regarding long-term safety.

### 3.6 Subgroup analysis

**Figure 4:**
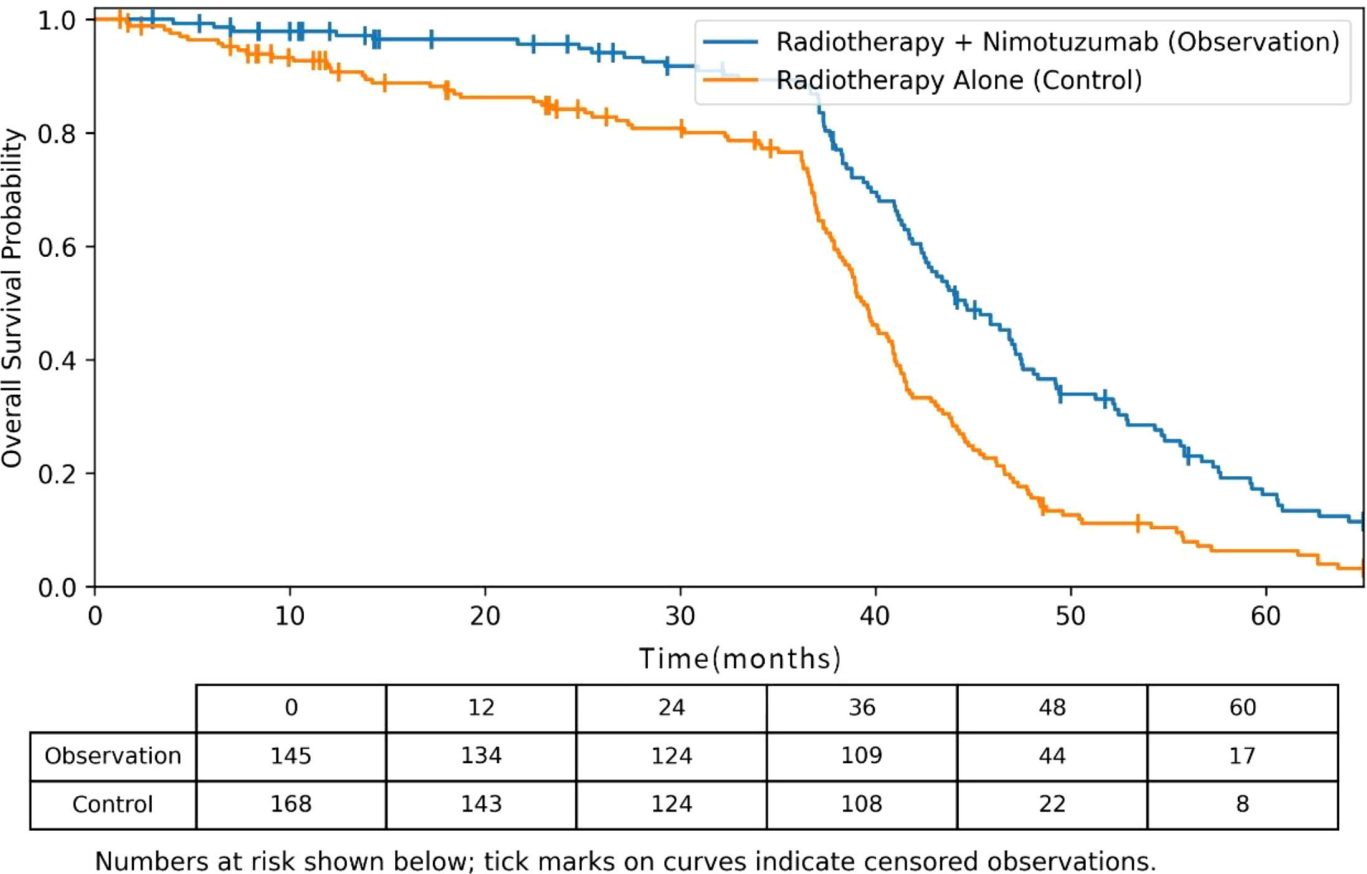
Kaplan-Meier curves for overall survival according to treatment group.

Kaplan–Meier curves for OS are presented in **Figure 4**. Censoring marks are indicated on the curves, and the numbers at risk are provided at predefined time points to enhance transparency. As shown, a clear separation between the two treatment groups is evident during the first 3–4 years of follow-up. However, the tail of the curves beyond approximately 50 months should be interpreted with caution due to the declining number of patients still under observation, particularly in the radiotherapy-alone group, which results in less stable survival estimates in the later follow-up period. The stepwise pattern observed in the late portion of the curves reflects the actual timing of events within a context of limited long-term follow-up rather than an artifact of the analytical method.

Subgroup analyses based on OS demonstrated that the survival benefit associated with nimotuzumab was particularly notable in patients with nasopharyngeal carcinoma (HR = 0.55, 95% CI: 0.36–0.85), those younger than 60 years (HR = 0.58, 95% CI: 0.38–0.89), and those with extensive lymph node involvement (N2–N3; HR = 0.61, 95% CI: 0.40–0.93). A significant interaction was observed between treatment modality and HNC type (NPC *vs*. non-NPC) (P for interaction = 0.032), suggesting a stronger treatment effect among NPC patients.

In contrast, no significant OS benefit was observed in the non-nasopharyngeal head and neck cancer subgroup (HR = 1.34, 95% CI: 0.82–2.19; P = 0.241). To address potential bias introduced by tumor-type heterogeneity, we performed a sensitivity analysis limited to NPC patients, which confirmed a consistent survival benefit with nimotuzumab within this population (HR = 0.55, 95% CI: 0.36–0.85). Together, these findings indicate that the overall survival advantage observed in the full cohort is predominantly driven by the NPC subgroup, and caution should be exercised when extrapolating these results to non-NPC head and neck cancers.

### 3.7 Correlation between depth of tumor response and survival outcomes

**Figure 5:**
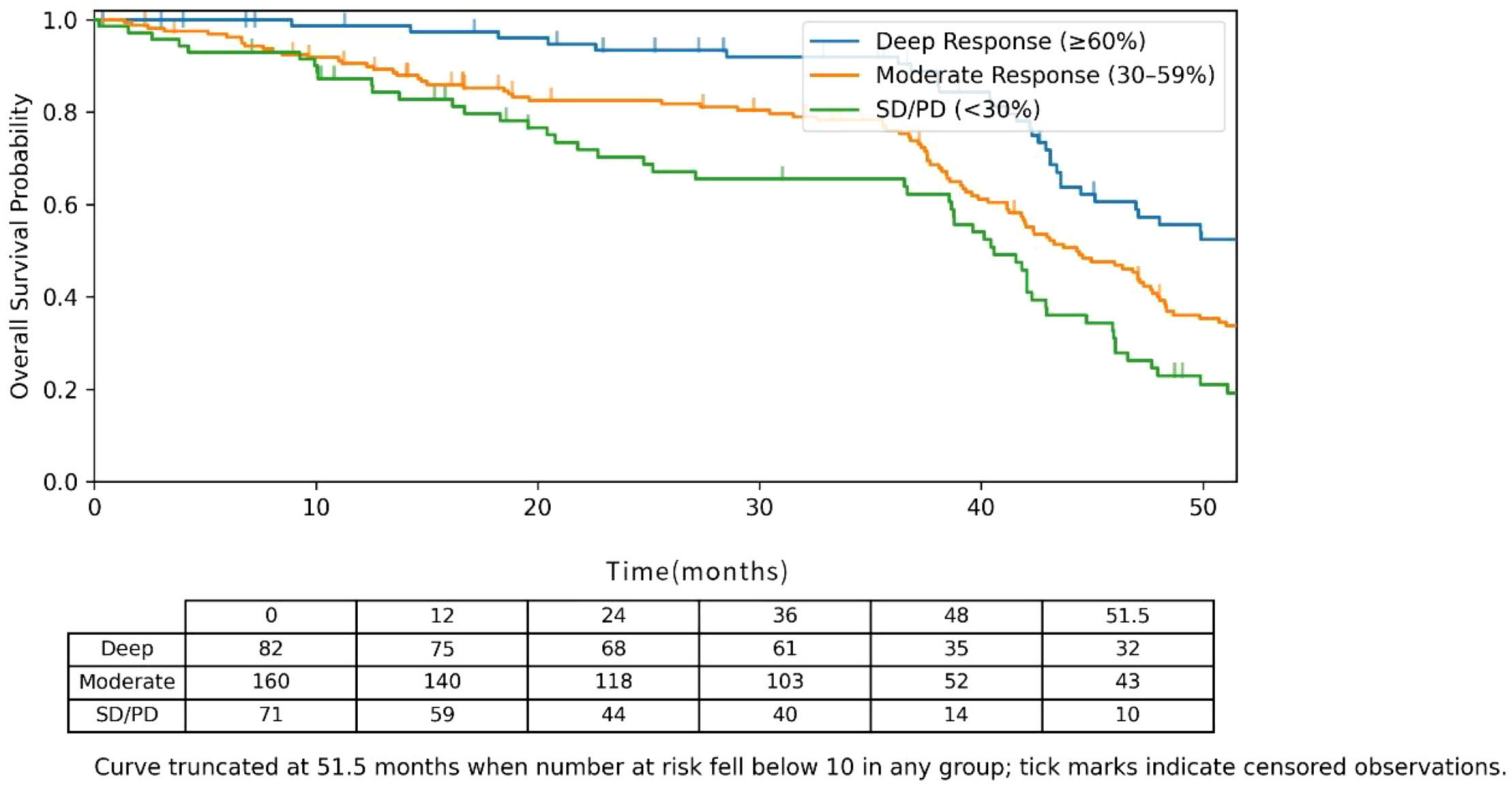
Kaplan-Meier overall survival (OS) curves by depth of tumor response groups.

We also examined the association between the degree of tumor shrinkage observed at the conclusion of post-induction radiotherapy—measured as the maximal percent reduction in the total diameter of target lesions from baseline—and subsequent long-term survival outcomes. Patients were divided into three groups based on response depth: Deep Response (shrinkage ≥60%), Moderate Response (shrinkage 30%–59%), and Stable Disease/Progressive Disease (SD/PD, shrinkage <30%). Kaplan-Meier analysis showed a significant difference in OS among the three groups (Log-rank P < 0.001). After adjusting in multivariate analysis, Deep Response remained an independent favorable predictor of OS (HR = 0.42, 95% CI: 0.25–0.71, P = 0.001). Notably, the proportion of patients achieving a Deep Response was significantly higher in the observation group compared to the control group (35.2% *vs*. 18.5%, P = 0.001), providing clinical evidence supporting the mechanism that “nimotuzumab improves survival by enhancing tumor response.” (**Figure 5**; **Table 5**).

**Table 5:** Multivariate Cox regression analysis of depth of tumor response as a predictor of overall survival.

Variable	HR	95% CI	P value
Depth of response group			
Deep Response (≥60%)	0.42	0.25 – 0.71	0.001
Moderate Response (30–59%)	0.78	0.51 – 1.19	0.251
Stable Disease/Progression (<30%)	1	(Reference)	–
**Age ≥60 years**	1.18	0.79 – 1.76	0.42
**Male**	1.05	0.67 – 1.66	0.825
**T Stage (T3–T4)**	2.01	1.25 – 3.24	0.004
**N Stage (N2–N3)**	1.5	0.95 – 2.37	0.083
**Clinical Stage (III–IV)**	2.65	1.58 – 4.45	<0.001
**Treatment Group (Observation)**	0.65	0.44 – 0.97	0.034

Bold values indicate statistical significance (P < 0.05).

## 4 Discussion

This retrospective study evaluated the clinical efficacy and safety of adding nimotuzumab to a sequential treatment protocol consisting of induction chemotherapy followed by radiotherapy in 313 patients with locally advanced HNC. The results demonstrate that the combined regimen not only yields superior short-term tumor responses and improved long-term survival, but also maintains a comparable incidence of severe treatment-related toxicities. Importantly, no excess treatment-related mortality was observed; the reported mortality rates reflect all-cause deaths during follow-up, with no fatalities attributed to acute toxicities from radiotherapy or nimotuzumab.

All patients in this study received standardized IMRT. The classic meta-analysis by Bourhis et al. highlighted that while hyper fractionated or accelerated radiotherapy improves local control compared with conventional techniques, it also increases the risk of normal tissue toxicity (14). Modern IMRT, through precise dose sculpting, enables optimal target coverage while maximally sparing organs at risk. This technological advancement was instrumental in ensuring an acceptable safety profile for our intensified treatment regimen, which combined induction chemotherapy with concurrent radiotherapy and targeted therapy. The absence of significant differences in typical radiotherapy-related toxicities—such as grade ≥3 radiation dermatitis and oral mucositis—between the observation and control groups may be partly explained by the widespread use of IMRT.

The role of systemic therapy in the multidisciplinary management of HNC has gained increasing recognition. The landmark MACH-NC meta-analysis by Pignon et al. established a clear survival benefit from integrating chemotherapy with radiotherapy, thereby solidifying concurrent chemoradiotherapy as the standard of care for locally advanced disease (15). Building on this foundation, our study explored the feasibility of incorporating a third therapeutic modality—targeted therapy—into the established paradigm of induction chemotherapy followed by radiotherapy. Our findings, which demonstrate superior complete response rates and overall survival in the observation group, suggest that EGFR-targeted therapy may confer an additional efficacy benefit beyond the synergy achieved with chemotherapy and radiotherapy. Notably, the absolute CR rates observed in our cohort were lower than those typically reported in homogeneous NPC series (often 80–95%), which is likely attributable to the inclusion of non-NPC HNC cases, the advanced disease stage of the cohort, and the relatively early timing of radiologic assessment.

Furthermore, the observation that partial response rates did not differ significantly between groups, while complete response rates and stable/progressive disease distributions did, aligns with a pattern in which nimotuzumab primarily converts non-responders into complete responders rather than incrementally increasing partial responses. All imaging evaluations were performed independently by two radiologists blinded to treatment assignment, with discrepancies resolved through consensus, thereby minimizing the possibility of systematic assessment bias. This pattern is consistent with the evolving trend in oncology toward multimodal, multi-targeted combination strategies.

The induction chemotherapy regimens used in this study included TP, DP, and TPF. The phase III trial by Vermorken et al. established the superior efficacy of the TPF regimen in advanced HNC, making it a standard induction option (4). Beyond reducing primary tumor size and downstaging disease, induction chemotherapy aims to eradicate micro metastases and enhance the efficacy of subsequent local therapy. The innovative aspect of our study lies in the combination of a radio sensitizing targeted agent with radiotherapy after effective induction chemotherapy, rather than proceeding with radiotherapy alone. This sequential intensification strategy may exert a synergistic effect: induction chemotherapy reduces tumor burden and may improve tumor perfusion, thereby facilitating the distribution and activity of subsequent nimotuzumab. Additionally, induction chemotherapy may modulate cell cycle or DNA repair pathways, potentially sensitizing tumor cells to the combined effects of radiotherapy and targeted therapy.

The combination of anti-EGFR targeted therapy with radiotherapy has a solid biological rationale. The pioneering study by Bonner et al. (16) was the first phase III trial to demonstrate that adding cetuximab to radiotherapy significantly improved overall survival in locally advanced HNC compared to radiotherapy alone, without exacerbating common radiotherapy toxicities. This provided high-level evidence for the application of anti-EGFR antibodies as radiosensitizers. Our study adopted this successful model but employed a different antibody (nimotuzumab) within a distinct treatment sequence (post-induction chemotherapy). Our results indicate that nimotuzumab also confers a significant survival benefit (HR = 0.62) within this specific treatment sequence, thereby extending the application of anti-EGFR therapy combined with radiotherapy to intensified regimens that include induction chemotherapy.

However, not all attempts to combine anti-EGFR antibodies with concurrent chemoradiotherapy have been successful. The RTOG 0522 study by Ang et al. (17) showed that adding cetuximab to concurrent chemoradiotherapy (cisplatin + accelerated radiotherapy) failed to improve survival and instead increased toxicity. This negative result underscores the need for careful balancing in treatment combination strategies. The “induction chemotherapy → radiotherapy + nimotuzumab” model used in our study temporally separates chemotherapy (induction phase) from the concurrent radiotherapy/targeted therapy phase (consolidation phase). This approach may avoid the toxicity overlap that can occur when multiple cytotoxic modalities (high-dose cisplatin and radiotherapy) are administered simultaneously with a targeted agent, thereby maintaining manageable safety while improving efficacy. This offers a novel perspective for optimizing combination treatment strategies.

Safety is a key determinant of a treatment regimen’s clinical applicability. The favorable tolerability profile of nimotuzumab combination therapy observed in our study is related to its unique pharmacological properties. Lacouture et al. (18) systematically described the mechanisms underlying skin toxicity from EGFR inhibitors, linking it to the intensity of EGFR signal inhibition in basal keratinocytes. Compared to cetuximab, nimotuzumab, due to its distinct binding epitope and more moderate affinity, is associated with a significantly lower incidence of severe skin toxicities (e.g., grade 3–4 acneiform rash) in clinical use (10). This safety advantage was also evident in our study, suggesting that this intensified regimen might be suitable for a broader patient population, including those with poorer tolerance to cetuximab-related toxicities.

The results of this study contribute new evidence to the treatment options for locally advanced HNC. The NCCN Clinical Practice Guidelines compiled by Pfister et al. (19) synthesize current best evidence to provide a framework for treatment decision-making, emphasizing the importance of developing individualized plans through multidisciplinary discussion. Our findings suggest that for certain patient subsets (e.g., those with nasopharyngeal carcinoma or younger age, as hinted by our subgroup analysis), an intensified strategy of radiotherapy combined with nimotuzumab following induction chemotherapy could be a viable consideration, particularly for patients who cannot tolerate the toxicity of concurrent chemoradiotherapy plus cetuximab, or in settings with relevant treatment expertise.

This study has certain limitations, primarily stemming from its single-center retrospective design, which inevitably introduces selection bias. Although multivariate analyses were performed to adjust for baseline differences—including age, pathological subtype, HNC type, and N stage—the potential influence of these imbalances on the results cannot be entirely ruled out. Additionally, while NPC constituted the majority of cases, the inclusion of a smaller number of non-NPC HNC patients, who differ in etiology, treatment sensitivity, and prognosis, may have introduced clinical and statistical heterogeneity. To address this, we conducted subgroup and sensitivity analyses restricted to the NPC population, which consistently demonstrated a survival benefit with nimotuzumab. These results suggest that the observed therapeutic advantage is likely driven primarily by effects within the NPC subgroup, and caution should be exercised in extrapolating these findings to non-NPC HNC. Despite these analytic efforts, residual confounding due to tumor-site heterogeneity cannot be fully excluded.

Further limiting the interpretability of our findings is the lack of molecular biomarker data—such as tumor EGFR expression levels and HPV/p16 status—which precludes a deeper mechanistic exploration of the differential treatment responses. Future prospective studies, ideally conducted with balanced baseline characteristics in more homogeneous patient populations and incorporating biomarker assessment, are needed to validate and refine these conclusions. Additionally, since patients received different induction chemotherapy regimens (TP, DP, and TPF) with potentially varying efficacy and toxicity profiles, these were included as covariates in multivariate models (including the logistic regression) to account for potential confounding effects; however, the relatively large number of covariates in relation to the number of events means that the logistic regression model remains statistically unstable and at risk of overfitting, so its estimates should be interpreted as hypothesis-generating rather than definitive. With respect to safety and patient-reported outcomes, late toxicity profiles and quality-of-life measures appeared comparable between the two groups in the subset with available data, suggesting that, within the observed follow-up window, the addition of nimotuzumab was not associated with a clear excess in treatment burden; however, the retrospective design, limited follow-up duration, incomplete capture of late events, and reliance on literature-based contextual data for very late toxicities mean that no firm conclusions regarding long-term safety or quality of life can be drawn from this study.

In conclusion, this study demonstrates that combining nimotuzumab with radiotherapy after induction chemotherapy can significantly enhance tumor response and improve overall survival in locally advanced HNC, without substantially increasing severe treatment-related acute toxicity. It is noteworthy that the treatment regimen demonstrated a manageable safety profile in the acute phase. Regarding long-term outcomes, while data on late toxicity and quality of life from our cohort are limited due to the retrospective nature and follow-up constraints, insights drawn from the existing literature suggest that this combination therapy warrants further prospective investigation to fully evaluate its late safety profile and impact on patient quality of life. These findings may offer a promising therapeutic strategy for optimizing outcomes in locally advanced nasopharyngeal carcinoma. Given the underrepresentation of non-NPC HNC in our cohort, however, these results should not be generalized to the broader head and neck cancer population without further validation. Future research should focus on prospective randomized controlled trials to confirm efficacy, the identification of predictive biomarkers to guide patient selection, and the optimization of treatment sequencing—ultimately aiming to maximize both survival benefit and quality of life for patients.

## Data Availability

The raw data supporting the conclusions of this article will be made available by the authors, without undue reservation.
