# Supplementary material for: Efficacy, safety, and survival outcomes of radiotherapy combined with nimotuzumab following induction chemotherapy in locally advanced head and neck cancer
Source: Front Oncol. 2026 Feb 18;16:1769718. doi: 10.3389/fonc.2026.1769718 (PMC12956683; doi:10.3389/fonc.2026.1769718)
Supplement: Supplementary file 1 [file Table1.docx]

**Table 1. Comparison of Baseline Characteristics between the Survival Group and Death Group (n, %)**

| **Characteristic** | **Category** | **Survival Group (n=257)** | **Death Group (n=56)** | **t/χ² Value** | **p Value** |
| --- | --- | --- | --- | --- | --- |
| **Gender, n (%)** | Male | 199 (77.43) | 43 (76.79) | 0.011 | 0.917 |
|  | Female | 58 (22.57) | 13 (23.21) |  |  |
| **Age Group, n (%)** | <60 years | 166 (64.59) | 36 (64.29) | 0.002 | 0.965 |
|  | ≥60 years | 91 (35.41) | 20 (35.71) |  |  |
| **Pathological Subtype, n (%)** | Undifferentiated non-keratinizing carcinoma | 192 (74.71) | 40 (71.43) | 0.934 | 0.817 |
|  | Differentiated non-keratinizing carcinoma | 22 (8.55) | 4 (7.14) |  |  |
|  | Keratinizing carcinoma | 5 (1.95) | 2 (3.57) |  |  |
|  | Squamous cell carcinoma | 38 (14.79) | 10 (17.86) |  |  |
| **HNC Type, n (%)** | Nasopharyngeal carcinoma | 221 (85.99) | 47 (83.93) | 0.159 | 0.69 |
|  | Non-nasopharyngeal carcinoma | 36 (14.01) | 9 (16.07) |  |  |
| **T Stage, n (%)** | T0 | 4 (1.56) | 1 (1.79) | 17.481 | **0.002** |
|  | T1 | 26 (10.11) | 12 (21.43) |  |  |
|  | T2 | 65 (25.29) | 8 (14.29) |  |  |
|  | T3 | 90 (35.02) | 9 (16.07) |  |  |
|  | T4 | 72 (28.02) | 26 (46.42) |  |  |
| **N Stage, n (%)** | N0 | 41 (15.95) | 2 (3.57) | 10.653 | **0.031** |
|  | N1 | 60 (23.35) | 15 (26.79) |  |  |
|  | N2 | 126 (49.03) | 27 (48.21) |  |  |
|  | N3 | 29 (11.28) | 12 (21.43) |  |  |
|  | N4 | 0 (0.00) | 0 (0.00) |  |  |
|  | N5 | 1 (0.39) | 0 (0.00) |  |  |
| **M Stage, n (%)** | M0 | 225 (87.55) | 49 (87.50) | 0 | 0.992 |
|  | M1 | 32 (12.45) | 7 (12.50) |  |  |
| **Clinical Stage, n (%)** | I | 7 (2.72)¹ | 2 (3.57) | 10.096 | **0.018** |
|  | II | 22 (8.56) | 8 (14.29) |  |  |
|  | III | 122 (47.47) | 14 (25.00) |  |  |
|  | IV | 106 (41.25) | 32 (57.14) |  |  |
| **Treatment Modality** | Radiotherapy + Nimotuzumab after IC | 126 (49.03) | 19 (33.93) | 4.216 | **0.04** |
|  | Radiotherapy after IC | 131 (50.97) | 37 (66.07) |  |  |

**Table 2. Variable Assignment for Logistic Regression**

| **Variable** | **Variable Name** | **Assignment Method** |
| --- | --- | --- |
| Y | Survival Prognosis | 1 = Survival / 0 = Death |
| X1 | T Stage | T Stage (T0): T0 = 1, Non-T0 = 0 |
|  |  | T Stage (T1): T1 = 1, Non-T1 = 0 |
|  |  | T Stage (T2): T2 = 1, Non-T2 = 0 |
|  |  | T Stage (T3): T3 = 1, Non-T3 = 0 |
|  |  | T Stage (T4): T4 = 1, Non-T4 = 0 |
| X2 | N Stage | N Stage (N0): N0 = 1, Non-N0 = 0 |
|  |  | N Stage (N1): N1 = 1, Non-N1 = 0 |
|  |  | N Stage (N2): N2 = 1, Non-N2 = 0 |
|  |  | N Stage (N3): N3 = 1, Non-N3 = 0 |
|  |  | N Stage (N4): N4 = 1, Non-N4 = 0 |
|  |  | N Stage (N5): N5 = 1, Non-N5 = 0 |
| X3 | Clinical Stage | Clinical Stage (I): Stage I = 1, Non-Stage I = 0 |
|  |  | Clinical Stage (II): Stage II = 1, Non-Stage II = 0 |
|  |  | Clinical Stage (III): Stage III = 1, Non-Stage III = 0 |
|  |  | Clinical Stage (IV): Stage IV = 1, Non-Stage IV = 0 |
| X4 | Treatment Method | 1 = Radiotherapy + Nimotuzumab after IC, 2 = Radiotherapy after IC |

**Table 3. Analysis of Factors Influencing Survival (Binary Logistic Regression)**

| **Variable** | **Variable Name** | **Category** | **B** | **S.E.** | **df** | **Wald** | **Sig.** | **Exp(B)** |
| --- | --- | --- | --- | --- | --- | --- | --- | --- |
| **X1** | T Stage | T0 | 0.501 | 1.43 | 1 | 0.123 | 0.726 | 1.65 |
|  |  | T1 | 0.498 | 0.605 | 1 | 0.676 | 0.411 | 1.645 |
|  |  | T2 | 1.91 | 0.665 | 1 | 8.255 | **0.004** | 6.752 |
|  |  | T3 | 1.629 | 0.592 | 1 | 7.583 | **0.006** | 5.101 |
|  |  | T4 | (Reference) |  | 0 |  |  |  |
| **X2** | N Stage | N0 | -18.324 | 40189.9 | 1 | 0 | 1 | 0 |
|  |  | N1 | -20.377 | 40189.9 | 1 | 0 | 1 | 0 |
|  |  | N2 | -20.741 | 40189.9 | 1 | 0 | 1 | 0 |
|  |  | N3 | -21.761 | 40189.9 | 1 | 0 | 1 | 0 |
|  |  | N4 | (Reference) |  | 0 |  |  |  |
| **X3** | Clinical Stage | I | -1.38 | 1.064 | 1 | 1.683 | 0.194 | 0.252 |
|  |  | II | -1.837 | 0.781 | 1 | 5.541 | **0.019** | 0.159 |
|  |  | III | -0.149 | 0.562 | 1 | 0.07 | 0.791 | 0.862 |
|  |  | IV | (Reference) |  | 0 |  |  |  |
| **X4** | Treatment Method |  | -0.804 | 0.345 | 1 | 5.441 | **0.02** | 0.448 |
